# Comparing the effect of diode laser against acyclovir cream for the treatment of herpes labialis

**DOI:** 10.4317/jced.53679

**Published:** 2017-06-01

**Authors:** Marieh Honarmand, Leila Farhadmollashahi, Ehsan Vosoughirahbar

**Affiliations:** 1 Associated Professor, Oral and Dental Disease Research Center, Department of Oral Medicine, School of Dentistry, Zahedan University of Medical Sciences, Zahedan, Iran; 2 Associated professor, Oral and Dental Disease Research Center, Department of Oral Medicine, School of Dentistry, Zahedan University of Medical Sciences, Zahedan, Iran; 3 Postgraduate Student, Dep of Oral Medicine, School of Dentistry, Zahedan University of Medical Science, Zahedan, Iran

## Abstract

**Background:**

Recently alternative therapies such as the use of diode laser therapy have been introduced for recurrent herpes labial infection. The aim of this study was to evaluate the effectiveness of diode laser for treatment of recurrent herpes labialis.

**Material and Methods:**

This was single-blind randomized clinical trial to evaluate the efficacy of diode laser for the treatment of recurrent herpes labial. In total, 60 patients whit recurrent herpes simplex labialis were selected and randomly divided in to three groups. 20 patients received treatment whit diode laser (at a wavelength of 870 nm, energy density 4.5 j/cm2), 20 patients were treated with acyclovir cream 5%, 20 patients received treatment with laser-off (placebo). The end point was lesions crusting. Data analyzed by Tukey HSD Test and One-way ANOVA (at a significance level of 0.05) in SPSS-20 software.

**Results:**

The mean length of recovery time (day) in the laser, off laser, and acyclovir groups was 2.20±0.41, 4.30±1.03, and 3.4±1.142, respectively. There is a significant difference between three groups in this regard (*P*<0.0001). The mean duration of pain (day) was 1.35±0.74, 2.65±1.27, and 2.30±0.92 for laser, off laser, and acyclovir groups, respectively (*P*<0.0001).

**Conclusions:**

Treatment with diode laser reduced the length of recovery time and pain severity faster than treatment with acyclovir cream.

** Key words:**Recurrent herpes labial, Acyclovir, Low level laser therapy.

## Introduction

Recurrent herpes labialis (RHL) occurs in 20% to 40% of young adult population. The lesions have prodromal symptoms, including itching, tingling or burning in 50% of cases and eventually develop papules, vesicles, ulcers, and crust. The pain often exists during the first two days (1). Internal or external stimuli such as stress, immunosuppression, high fever, trauma, and ultraviolet light can trigger recurrences (2). The lesions usually resolve within 7 to 10 days (3).

Although RHL is a self-limiting condition, the use of topical antiviral medications reduces viral shedding and infectivity. These agents also decrease pain level, lesion size and duration of symptoms (1). Antiviral medications such as acyclovir cream 5% and docosanol cream 10% can be beneficial if initiated during the onset of lesions. Since these agents have relatively a short half-life, they should be used several times throughout the day (1). Risk of drug nephrotoxicity should be considered for systemic administration (4,5).

Low level laser therapy (LLLT) has been considered as a possible treatment for herpes labialis in recent years. The laser phototherapy has analgesic and anti-inflammatory properties and stimulates tissue regeneration, fibroblast proliferation and neo-vascularization potential (6).

Many studies have examined the influence of LLLT wavelengths on herpes labialis.

Dougal and Lee (2) assigned 87 patients with herpes labialis randomly in two groups. They used low level laser, 1072 nm, for the experimental group. The control group was treated with the laser turned off. The experimental group showed a significant reduction in healing time.

Muñoz Sanchez *et al.* (7) conducted a study in 2012 in which they compared the effect of LLLT, 670 nm, on herpes labialis with acyclovir. They reported that LLLT was an effective therapy with no side effects.

This study aimed to evaluate the effect of Low level laser therapy on the treatment of patients with herpes labialis compared to acyclovir cream.

## Material and Methods

This single-blind randomized clinical trial was carried out on 60 patients with herpes labialis referring to the oral medicine department of Zahedan university of medical science, Before entering the study, patients were informed regarding the purpose of this study and provided a signed consent to participate in this study. The protocol was approved by Ethical Committee of Zahedan University of medical science (code 7030). The participants had no systemic diseases or oral lesions and did not take other medications during the study. All patients had herpes labialis lesions developed 0-36 hours before the study.

The patients were randomly divided into three 20-member groups. The three groups were matched for age and sex. The first group was treated using acyclovir cream (5%) 5 times per day.

The second group was treated with diode laser [(LO7 probe, Mustang 2000, Russia), wavelength 870nm, energy density 4.5j/cm2, frequency 600Hz and maximum pulse radiation power 80W for a minute/ day]. The tip of the probe was held 6-8mm from the lesion.

In the third group, laser therapy was performed using a turned-off device. It should be noted that we used the protective eyewear for the safety of patients. Patients were not informed about the possible benefits of laser treatment.

The treatment was performed by an oral medicine specialist, and the patients were examined on daily basis using a dental chair light source. Intended parameters included lesion size, pain level and length of time for crust development. The examination was done by an oral medicine assistant who was blinded to treatment.

The lesion size was determined by measuring the two main diameters of the lesion in square millimeters using a transparent graph paper.

The pain intensity was evaluated based on the visual analogue scale (VAS) before treatment and at each follow-up session. According to this scale, 0 represented no pain and 10 represented the most severe pain ever experienced. Crust formation and erythema resolution were healing signs. The aforementioned information was recorded in relevant data sheets.

Data analyzed by Tukey HSD Test and One-way ANOVA (at a significance level of 0.05) in SPSS-20 software.

## Results

This study aimed to compare the effect of diode laser on herpes labialis with acyclovir. Sixty participants were divided into three groups. The laser group consisted of 15 males (75%) and 5 females (25%) with the mean age of 31.30±10.032. The turned-off laser group consisted of 15 males (75%) and 5 females (25%) with the mean age of 32.85±6.808. The acyclovir-treated group consisted of 14 males (70%) and 6 females (30%) with the mean age of 31.35±6.862. The mean age and the sex distribution were similar in all three groups.

The average area of the lesion in laser, turned-off laser and acyclovir-treated groups was 25.55±15.99mm2, 25.75±12.74mm2, and 25.90±15.05mm2, respectively. There was no statistically significant difference between these groups (*p*=0.997). In addition, the pain level based on VAS was 3.65±2.581 in laser group, 3.50±2.705 in turned-off laser group, and 3.50±2.065 in acyclovir-treated group before the treatment, and no statistically significant difference was observed among the three groups (*p*=0.976).

One-way ANOVA test demonstrated that there was a statistically significant difference among the three groups during the treat-ment process in terms of pain intensity ( Table 1). Two-group assessment using Tukey HSD showed a significant difference among the groups in terms of pain intensity.

**Table 1:**
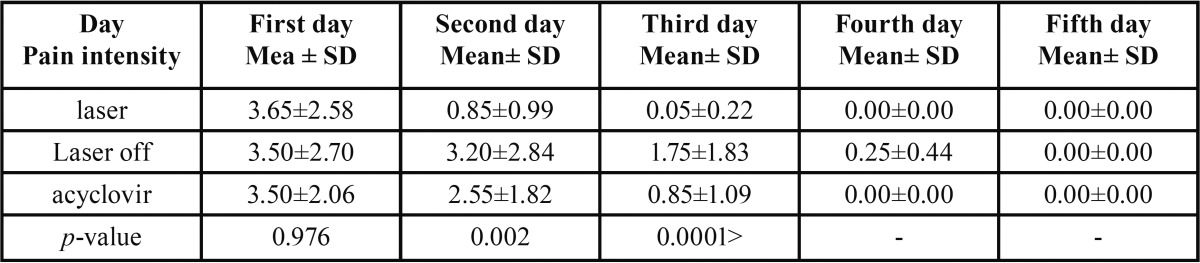
Pain intensity in the three groups on different days of treatment.

One-way ANOVA test for the mean and standard deviation of lesion area showed that there was a statistically significant difference among the three groups ( Table 2). Two-group assessment using Tukey HSD showed a significant difference among the groups in terms of lesion area.  Table 2 demonstrates the mean and standard deviation of lesion area in the three evaluated groups on different days of treatment.

**Table 2:**
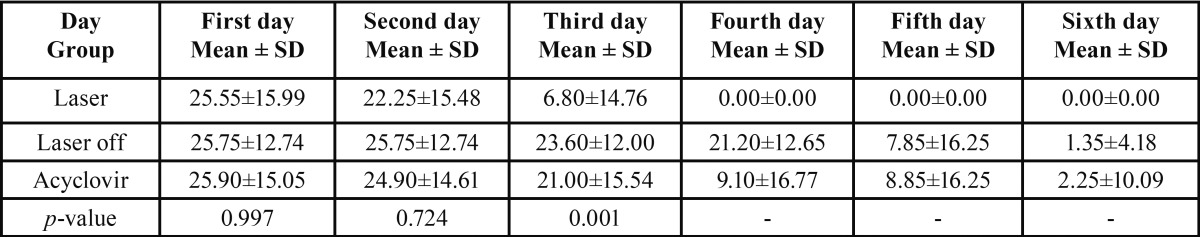
Mean and standard deviation of lesion area in the three evaluated groups on different days of treatment.

It is noteworthy that in the turned-off laser group on the fifth and sixth days, only 7 and 2 patients were still under treatment, respectively. The other participants’ lesions had developed crust on the fourth day.

In the acyclovir group, on the fourth, fifth, and sixth days, only 5, 5, and 1 patient were still under treatment, respectively considering their lesion, and the other patients of this group had recovered during the first three days of treatment.

As  Table 3 shows, the mean recovery time (day) was 2.20±0.41 in the laser group, 4.30±1.03 in the turned-off laser group and 3.4±1.142 in the acyclovir group, which showed a statistically significant difference (*p*<0.0001).

**Table 3:**
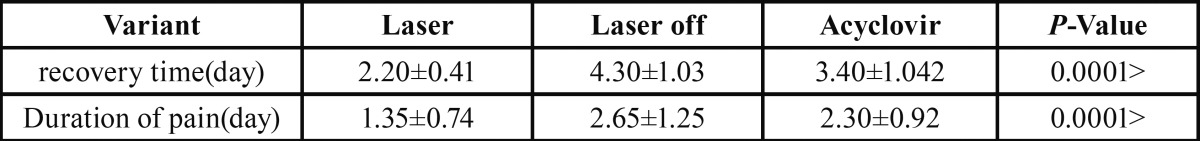
Comparison of clinical characteristics in three groups.

In addition, the mean treatment duration of pain in terms of days in the three groups of laser, turned-off laser, and acyclovir was 1.35±0.74, 2.65±1.27, and 2.30±0.92, respectively, which showed a statistically significant difference (*p*<0.0001). Tukey HSD test also showed that the two-group difference was significant.

## Discussion

Recurrent herpes labialis infection occurs in more than one fifth of young adult population. Transmission may occur via contact with an infected person. The virus remains in a latent state in neural tissues, and internal or external stimuli such as stress, immunosuppression, high fever, trauma, and ultraviolet light can trigger recurrences (2).

Herpes labialis not only causes pain and discomfort, but also periods of feeling sick during the infection which can affect a person’s work and social activities (8).

Effective antiviral medications on herpes labialis such as acyclovir have the most influence while using at the onset of the lesion; however, late treatment is associated with prolonged symptoms and persistent lesions. In addition, these agents should be administered several times on consecutive days that can have even sometimes interactions with other medications (2).

Acyclovir is an effective treatment for herpes labialis with a short half-life, whereas famciclovir and valacyclovir have longer half-lives; however, they are not available as topical agents. Some acyclovir and famciclovir-resistant strains of HSV have emerged mainly due to cavalier use of these agents. In addition, systemic medications can increase the risk of nephrotoxicity in case the patient gets dehydrated (4,5).

In general, these difficulties with conventional medications have directed investigators toward other therapeutic approaches. Attention has been given to laser therapy as a new treatment method for herpes labialis.

Low level laser therapy has physiological effects such as anti-inflammatory, analgesic and healing-stimulating characteristics (6).

Many studies have evaluated different wavelengths of low level laser on herpes labialis (7-10).

This study also aimed to evaluate the effect of diode laser on treatment of patients with herpes labialis compared with acyclovir. The low level laser therapy (LLLT) significantly decreased the healing time, and pain intensity compared to acyclovir and turned-off laser groups, which is consistent with previous studies.

In a study conducted in 2013 by Dougal and Lee (2), the effect of diode laser (1072 nm) on herpes labialis was assessed. The results showed that the crust time and healing time for herpes labialis decreased in the laser group compared to the control group.

In a study conducted in 2006 by Hargate *et al.* (9), the effect of diode laser (1072nm) on herpes labialis was assessed. The results showed that the mean crust time was two days for the experimental group and 2.88 days for the control one. The mean healing time was 6.33 and 9.40 days for the experimental and control groups, respectively.

Eduardo *et al.* (10) conducted a study in 2012 with a 3 year follow-up which showed that LLLT as a prophylactic treatment for recurrent herpes labialis not only reduced lesion frequency, but also made healing quicker and symptoms less intense.

De Carvalho *et al.* (3) (2010) reported that Ga-Al-As laser (780nm) (one weekly session, 10 sessions in total) reduced the lesion size and inflammation.

Marotti *et al.* (11) conducted a study in 2009 using diode laser, 660nm, to treat patients with herpes labialis in the vesicle stage. They suggested that laser therapy reduced recurrence rates, relieved symptoms and has no side effects.

Several mechanisms have been proposed for therapeutic effects of LLLT. Laser induces more ATP production in mitochondria and reduces cellular oxygen consumption. Levels of serotonin and endorphins increase, prostaglandin production decreases, and cytokine and growth factor expression promotes which eventually causes inflammation reduction and healing process promotion. In addition, increased skin blood circulation, lymphatic drainage and hyperpolarization reduce edema (12).

Based on the results of this study and other investigations, it can be concluded that low level laser therapy reduces the length of recovery time and pain level and promotes the healing process compared to acyclovir.

## References

[1] Arain N, Paravastu SC, Arain MA (2015). Effectiveness of topical corticosteroids in addition to antiviral therapy in the management of recurrent herpes labialis: a systematic review and meta-analysis. BMC Infect Dis.

[2] Dougal G, Lee SY (2013). Evaluation of the efficacy of low-level light therapy using 1072 nm infrared light for the treatment of herpes simplex labialis. Clin Exp Dermatol.

[3] de Carvalho RR, de Paula Eduardo F, Ramalho KM, Antunes JL, Bezinelli LM, de Magalhães MH (2010). Effect of laser phototherapy on recurring herpes labialis prevention: an in vivo study. Lasers Med Sci.

[4] Izzedine H, Launay-Vacher V, Deray G (2005). Antiviral drug-induced nephrotoxicity. Am J Kidney Dis.

[5] Opstelten W, Neven AK, Eekhof J (2008). Treatment and prevention of herpes labialis. Can Fam Physician.

[6] Wagner VP, Meurer L, Martins MA, Danilevicz CK, Magnusson AS, Marques MM (2013). Influence of different energy densities of laser phototherapy on oral wound healing. J Biomed Opt.

[7] Muñoz Sanchez PJ, Capote Femenías JL, Díaz Tejeda A, Tunér J (2012). The effect of 670-nm low laser therapy on herpes simplex type 1. Photomed Laser Surg.

[8] Arduino PG, Porter SR (2008). Herpes Simplex Virus Type 1 infection: overview on relevant clinico-pathological features. J Oral Pathol Med.

[9] Hargate G (2006). A randomised double-blind study comparing the effect of 1072-nm light against placebo for the treatment of herpes labialis. Clin Exp Dermatol.

[10] Eduardo Cde P, Bezinelli LM, Eduardo Fde P, da Graça Lopes RM, Ramalho KM, Bello-Silva MS (2012). Prevention of recurrent herpes labialis outbreaks through low-intensity laser therapy: a clinical protocol with 3-year follow-up. Lasers Med Sci.

[11] Marotti J, Aranha AC, Eduardo Cde P, Ribeiro MS (2009). Photodynamic therapy can be effective as a treatment for herpes simplex labialis. Photomed Laser Surg.

[12] Cotler HB, Chow RT, Hamblin MR, Carroll J (2015). The Use of Low Level Laser Therapy (LLLT) For Musculoskeletal Pain. MOJ Orthop Rheumatol.

